# Distance professional qualification to promote adequate and healthy eating: factors associated with dropout

**DOI:** 10.1590/S2237-96222025v34e20240338.en

**Published:** 2025-08-08

**Authors:** Mariana Souza Lopes, Izabela Santana Sathler, Cheila de Sousa Jardim Soares, Nathália Luíza Ferreira, Aline Cristine Souza Lopes

**Affiliations:** 1 Universidade Federal da Paraíba, Centro de Ciências da Saúde, Departamento de Nutrição, PB, Brazil; 2 Universidade Federal de Minas Gerais, Escola de Enfermagem, Departamento de Nutrição, MG, Brazil; 3 Universidade Federal de Lavras, Faculdade de Ciências da Saúde, MG, Brazil

**Keywords:** Brazilian National Health System, Diet, Healthy, Education, Distance, Education, Continuing, Cross-Sectional Studies, Sistema Único de Salud, Dieta Saludable, Educación a Distancia, Educación Continua, Estudios Transversales

## Abstract

**Objective:**

To analyze factors associated with dropout from a distance professional qualification course on promoting adequate and healthy eating in Brazil.

**Methods:**

This is a cross-sectional study with professionals from the Brazilian National Health System (*Sistema Único de Saúde* - SUS) enrolled in a distance learning course, provided between 2019 and 2020, based on Ministry of Health educational materials. Sociodemographic and professional data associated with dropout from the course were assessed, using multiple logistic regression (p-value<0.05).

**Results:**

Dropout prevalence was 30.6%. It was was associated with living in the Northern macroregion (p-value 0.009) and the Northeast macroregion (p-value<0.001), being a nurse (p-value<0.001), having lower levels of higher education (degree: p-value 0.004; specialization/residency: p-value 0.025), having worked in the SUS for longer (p-value 0.004), having taken the course using a cell phone or tablet (p-value 0.024), having previous training on adequate and healthy eating (p-value 0.007) and not having previously used the course materials (p-value 0.021).

**Conclusion:**

Dropout was greater among nurses, professionals from the North and Northeast macroregions, those with a lower level of higher education, longer experience in the SUS, and among those who accessed the course mainly via cell phone or tablet, those who had previously participated in professional training on the theme and those who had not previously used the materials presented during the course. Changing these factors requires institutional support, with integration of the country’s federative units, aiming to overcome local challenges faced by professionals in completing continuing education activities.

## Resumen

**Objetivo:**

Analizar los factores asociados al abandono de un curso de cualificación profesional a distancia para promover alimentación adecuada y saludable en Brasil.

**Métodos:**

Se trata de un estudio transversal con profesionales del Sistema Único de Salud (SUS) matriculados en un curso a distancia, ofrecido entre 2019 y 2020, con base en materiales del Ministerio de Salud. Se evaluaron datos sociodemográficos y profesionales asociados al abandono, utilizando regresión logística múltiple (valor de p <0,05).

**Resultados:**

La prevalencia del abandono fue del 30,6%. La evasión estaba asociada con vivir en las macrorregiones Norte (valor p 0,009) y Nordeste (valor p<0,001), ser enfermero (valor p<0,001), tener menores niveles de formación (graduación: valor p 0,004; especialización/residencia: p-valor 0,025), haber trabajado durante más tiempo en el SUS (p-valor 0,004), haber completado el curso utilizando un teléfono celular o tableta. (p-valor 0,024), tener cualificación previa en alimentación adecuada y saludable (p-valor 0,007) y no haber utilizado previamente los materiales del curso (p-valor 0,021).

**Conclusión**

La evasión estaba asociada con residir entre enfermeros, profesionales de las macrorregiones Norte y Nordeste, con menor nivel de formación, mayor experiencia en el SUS, y entre quienes accedieron al curso principalmente por teléfono celular o tableta, que habían participado previamente en calificación profesional sobre el tema y que no habían utilizado los materiales utilizados en el curso. Modificar estos factores requiere apoyo institucional, con integración de diferentes entidades federadas, con el objetivo de superar los desafíos locales que enfrentan los profesionales para realizar actividades de educación continua.

**Ethical aspects**

This research respected ethical principles, having obtained the following approval data::

Research Ethics Committee: Universidade Federal de Minas Gerais

Opinion number: 4294291

Approval date: 23/9/2020

Certificate of Submission for Ethical Appraisal: 93992418.0.0000.5149

Informed Consent Form: Signed by all participants.

## Introduction

Promotion of adequate and healthy eating can be found in several different Brazilian normative frameworks, such as the National Food and Nutrition Policy and the National Health Promotion Policy. These consist of strategies to encourage biologically and socioculturally appropriate eating practices, which contribute to improve the population’s quality of life, nutrition and health (1). The Food Guide for the Brazilian Population is used in the Brazilian National Health System (*Sistema Único de Saúde* - SUS) as a basis for actions to promote adequate and healthy eating. It is an innovative instrument that takes into consideration the different dimensions of nutrition (2). 

Materials produced by the Brazilian Ministry of Health stand out among several initiatives aimed at supporting the implementation of the Food Guide in the SUS, in particular the: “Instructions for froup-based methodology for food and nutrition actions in Primary Health Care” (3) and their supporting materials. In addition to the provision of instructional materials, health professionals need to be trained to use them in their work (4). As such, the Ministry of Health has invested in continuing health education actions aligned with SUS guidelines (5).

Carrying out ongoing health education actions in a country of continental dimensions such as Brazil and within the context of different SUS realities requires comprehensive and flexible strategies, such as distance learning (6). However, this modality has limitations, with emphasis on its high dropout percentages, which have ranged from 30.0% to 75.0% (7,8). These high dropout rates can lead to materials and human resources being underutilized, in addition to compromising the qualification of health professionals (9), which highlights the importance of investigating them in greater depth. 

Factors such as insufficient technical and didactic support, technological difficulties, course quality, lack of social interaction, heavy work or study activity loads, and student satisfaction and motivation are among the main aspects related to non-completion of distance learning courses (10,11,12). The data provided by this research will be able to support health service managers and contribute to the design of more robust and effective strategies for student retention and certification. This study aimed to analyze factors associated with dropout from distance professional training activities aimed at SUS professionals promoting adequate and healthy eating across Brazil. 

## Methods

### Study design 

This is a cross-sectional study that analyzed data on health professionals who either completed or did not complete a professional qualification course aimed at developing collective activities in the SUS, called “Professional qualification course on promoting adequate and healthy eating in Primary Health Care” 

### Background

The course took place online between May 2019 and November 2020 and had three separate classes. The course was designed by a research group in partnership with the General Food and Nutrition Coordination Sector, a division of the Department of Prevention and Health Promotion, part of the Ministry of Health’s Primary Health Care Secretariat. The course was made available via the Telehealth (Telessaúde) platform, using the Moodle virtual learning environment. 

The Ministry of Health requested and supported the training course, mainly due to the need to improve the performance of SUS health professionals, especially within Primary Health Care, regarding actions to promote adequate and healthy eating.

The activity was based on the Food Guide for the Brazilian Population (2), the “Instructions for froup-based methodology for food and nutrition actions in Primary Health Care” (3) and its support materials entitled: “Demystifying doubts about food and nutrition: support material for health professionals” (13) and “In the kitchen with fruit and vegetables” (14).

The course’s political pedagogical project was discussed with pedagogues from the Telehealth team and approved by the Ministry of Health. It consisted of six modules, 11 video classes, five short videos, five discussion forums and seven practical activities. Module I presented the Brazilian food and nutrition scenario. Module II discussed the concept of adequate and healthy eating based on the Food Guide (2). Module III addressed concepts of continuing health education and food and nutritional education. Modules IV, V and VI covered the contents and applications of the group methodology for promoting adequate and healthy eating (3).

It was a 30-hour course, intended to be taken over two and a half months at the least. In the case of Classes 2 and 3, the course duration was extended to 11 months due to the coronavirus pandemic (COVID-19). The course was advertised via a short video sent by the research group’s emails, messaging applications and social networks, as well as by Telehealth and the Ministry of Health itself. The Ministry of Health also sent invitation letters to state and municipal health service managers.

### Participants

Health professionals with higher education qualifications, from all of Brazil’s macroregions, and who worked or developed actions in the SUS were eligible to enroll in the course. This included nutritionists, nurses, doctors, psychologists, social workers and speech therapists. 

Enrollment in Classes 1 and 2 took place firstly in the North, Northeast and Midwest macroregions in order to promote equity of access. Enrollment remained open for approximately 20 days and was validated according to the National Registry of Health Establishments. Class 3 included students nominated by the state health departments participating in the Call to Address and Control Obesity in the SUS, aimed at enhancing adequate and healthy nutrition promotion actions in across the country. 

The inclusion criteria for participation in this study were: being a student in Classes 1, 2 or 3, who answered the evaluation instrument sent three months after the end of the course. Participants who did not answer questions that identified them, such as full name and email, were excluded.

### Study size 

We analyzed all students enrolled in the course, regardless of whether they completed it or not, and who met the inclusion and exclusion criteria.

### Data sources and measurement 

Data relating to the course participants were obtained at two different times: at the beginning of the course and three months after the end of the course. At the beginning of the course, participants filled out a registration form and answered a questionnaire regarding sociodemographic data, information on professional training and performance, previous participation in professional qualification activities on the topic and use of materials presented during the course (2,3,13,14). 

After three months, a questionnaire in Google Forms format was sent by email. Up to three reminders were sent over three consecutive weeks with the aim of obtaining replies. This questionnaire included questions regarding the applicability and relevance of the course content and materials in professional practice, the carrying out of proposed educational activities, the dissemination of educational materials and possible barriers to completing the course.

### Variables

The outcome variable of this study was the “dropout from the professional qualification activity”, identified by comparing the lists of course completion certificates issued with the lists of enrolled students.

The following covariables were analyzed.

- Sociodemographic: sex (female, male), age (years), age group (years: 19-30, 31-40, 41-70) and Brazilian macroregion (Southeast, South, Midwest and Federal District, Northeast, North).
- Professional qualifications and work: profession (nutritionist, nurse, other – community health agent, social worker, undergraduate student, biologist, dental surgeon, physical education professional, physiotherapist, journalist, psychologist, nursing technician), time since degree graduation (years), level of higher education (degree, specialization, residency, master’s degree, doctorate), length of service in the SUS (years) and working hours (hours/week).
- Professional qualification and access to materials for promoting adequate and healthy eating: main form of access to the course (computer, cell phone or tablet), prior participation in professional qualification activities on promoting adequate and healthy eating (no, yes), prior use of the materials used in the course (no, yes), course class attended (1, 2, 3), main reason for dropping out (lack of time, operational difficulties on the platform, inadequate infrastructure, increased workload, qualification content, other, no obstacles), and, for Classes 2 and 3, whether the COVID-19 pandemic interfered with taking the course (no, yes).

The quality of the course was not assessed in the case of those who did not complete it, as this assessment was one of the activities of the last module of the course. If the student had dropped out before accessing the last module, they would have been unable to access the assessment activity. Assessment of the course by students who did not complete it could have been biased and not equal to those who did complete it, as each participant might not have dropped out of the same module.

### Bias

To guarantee data control and quality, and adequate internal validity of the study, all instruments were pre-tested and adjusted according to the needs identified. The entire data collection process was supervised by a health professional, under the guidance of the researchers.

### Statistical methods 

The analyses were performed using Stata Statistical Software for Data Science 15.0, including descriptive analyses, with calculation of frequency distributions, measures of central tendency and dispersion. Categorical variables were presented in the form of relative frequencies, while continuous variables were presented as medians and interquartile ranges (P_25_ – P_75_). Data on those who completed and did not complete the course were compared using Pearson’s chi-square and Mann-Whitney statistical tests. Variables associated with the outcome that had a significance level lower than 20% were included in a multiple logistic regression model (p-value<0.05) and were presented as odds ratios (OR) and 95% confidence intervals (95%CI).

## Results

**Figure 1:**
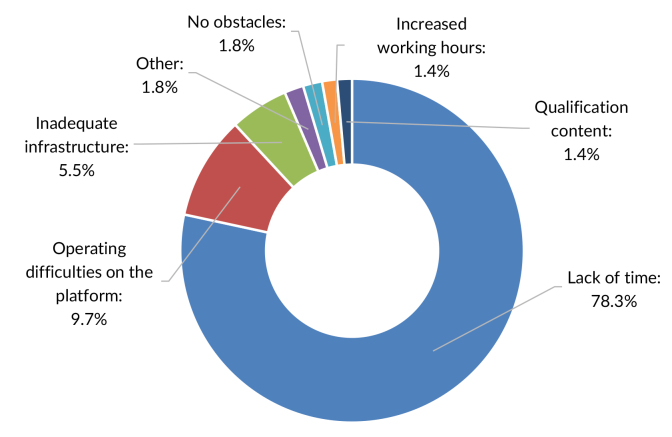
Main factors contributing to dropouts from the professional qualification course on promoting adequate and healthy eating in primary care. Brazil, 2019-2020 (n=486)

Of the students included in the study (n=818), 69.4% (n=568) completed the course. The dropout rate was 30.6% (n=250). As perceived by them, lack of time was the main obstacle to course completion (78.3%), followed by difficulties in operating the online platform (9.7%) and inadequate workplace infrastructure (5.5%) (Figure 1). 

**Figure 2:**
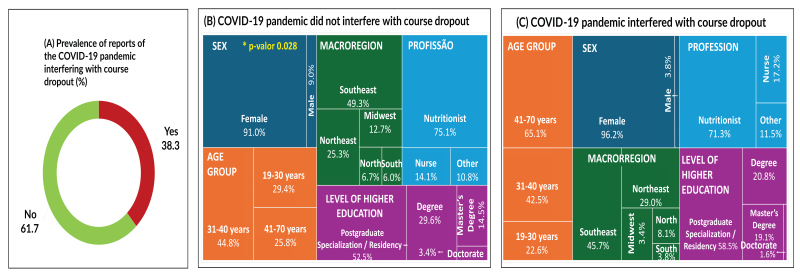
Influence of the COVID-19 pandemic on dropout from the professional qualification course on promoting adequate and healthy eating in primary care, and sociodemographic and professional training characteristics according to reported impact of the pandemic on course completion. Brazil, 2020 (n=486)

With regard to Classes 2 and 3, 38.3% of students indicated that the COVID-19 pandemic was an obstacle to completing the course (Figure 2A), with a higher prevalence of females (96.2%), compared to those for whom the pandemic was not an obstacle (91.0%; p-value 0.028). There were no differences regarding macroregion, age group, profession and higher education level of the participants (p-value>0.05) (Figures 2B and 2C).

Students who dropped out of the professional qualification course, when compared to those who completed it, had higher median age (in years: 36; 31-43 versus 35; 30-40; p-value 0.009]. The proportion of dropout was also higher among professionals who worked in the Northeast macroregion (38.8% versus 24.1%; p-value<0.001) and the Northern macroregion (10.8% vs. 6.0%; p-value<0.001) (Table 1).

**Table 1:** Prevalence of sociodemographic and professional characteristics of students on the professional qualification course on promoting adequate and healthy eating in primary care. Brazil, 2019-2020 (n=818)

Variable	Total (n=818) (%)	Course completed (n=568) (%)	Course not completed (n=250) (%)	p-value
**Sex**				0.248
Female	93.8	93.5	94.4	
Male	6.2	6.5	5.6	
**Macroregion of the country**				<0.001
Southeast	42.8	46.7	34.0	
South	10.4	10.9	9.2	
Midwest/Federal District	10.8	12.3	7.2	
Northeast	28.6	24.1	38.8	
North	7.5	6.0	10.8	
Profession				<0.001
Nutritionist	75.1	79.5	65.0	
Nurse	18.3	12.1	32.5	
Other professional category	6.6	8.4	2.5	
**Level of higher education**				0.001
Degree	27.4	25.2	32.4	
Specialization/residency	56.9	56.1	58.7	
Master’s degree	14.0	16.2	8.9	
Doctorate	1.7	2.5	0.0	
**Median** (**percentiles 25**-75)				
Age	35 (30-41)	35 (30-40)	36 (31-43)	0.009
Time since degree graduation (years)	12 (8-17)	12 (7-17)	13 (8-17)	0.083
Time working in the Brazilian National Health System (years)	6 (3-12)	5 (2-11)	8 (3.5-14)	<0.001
Working hours (hours/week)	40 (30-40)	40 (30-40)	40 (30-40)	0.176

The highest prevalence rates identified among students who dropped out related to nurses (32.5% versus 12.1%; p-value<0.001), professionals who only had a degree (32.4% vs. 25.2%; p-value 0.001) and those who only had specialization/residency (58.7% versus 56.1%; p-value 0.001) as their maximum level of higher education, as well as those who had the highest median time working in the SUS (in years: 8; 3.5-14 vs. 5.0; 2-11; p-value<0.001), when compared to those who did not drop out and completed the course (Table 1).

**Figure 3:**
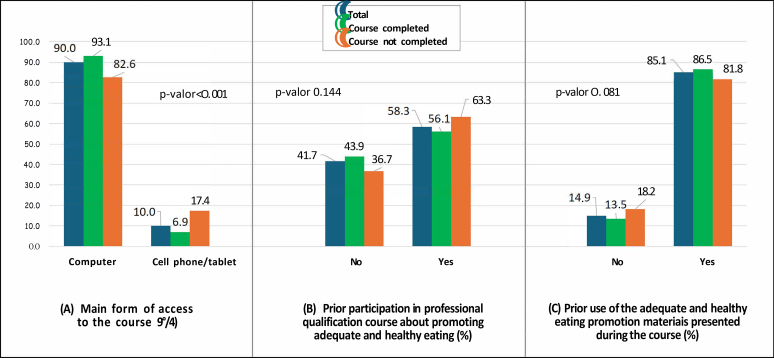
Access to the professional qualification course on promoting adequate and healthy eating in primary care and training to promote adequate and healthy eating. Brazil, 2019-2020 (n=818)

The preferred way of accessing the course was by computer (90.0%). There was a higher prevalence of cell phone or tablet use (17.4% vs. 6.9%; p-value<0.001) among students who dropped out. The majority of participants had already completed training on the topic (58.3%) and already used materials to promote adequate and healthy eating that were presented in the course (85.1%) (Figure 3)

In the multivariate analysis, there was greater likelihood of dropping out of professional qualification activities among professionals who worked in the Northern macroregion (OR 2.40; 95%CI 1.25; 4.63; p-value 0.009) and the Northeast macroregion (OR 2.16; 95%CI 1,44; 3.24; p-value<0.001), nurses (OR 2.80; 95%CI 1.80; 4.35; p-value <0.001), those who had lower levels of higher education (degree: OR 2.51; 95%CI 1.34; 4.70; p-value 0.004 and specialization/ residency: OR 1.92; 95%CI 1.08; 3.40; p-value 0.025); those who had worked for longer in the SUS (OR 1.04; 95%CI 1.01; 1.06; p-value 0.004), those who preferred to access the course via cell phone or tablet (OR 1.91; 95%CI 1.09; 3.34 ; p-value 0.024), those with prior participation in training courses on promoting adequate and healthy eating (OR 1.68; 95%CI 1.15; 2.46; p-value 0.007) and who had not previously used the materials presented during the course (OR 1.81; 95%CI 1.09; 2.98; p-value 0.021) (Table 2). 

**Table 2:** Crude and adjusted odds ratios (OR) and 95% confidence intervals (95%CI) generated in multivariate analysis of factors associated with dropout from the professional qualification course on promoting adequate and healthy eating in primary care. Brazil, 2019-2020 (n=818)

Variables	Crude OR (95%CI)	Adjusted OR (95%CI)	p-value
**Macroregion of the country**			
Southeast	1.00	1.00	-
South	1.16 (0.68; 1.98)	1.30 (0.72; 2.35)	0.381
Midwest/Federal District	0.80 (0.45; 1.42)	0.78 (0.40; 1.51)	0.465
North	2.46 (1.41; 4.34)	2.40 (1.25; 4.63)	0.009
Northeast	2.21 (1.54; 3.15)	2.16 (1.44; 3.24)	<0.001
Profession			
Nutritionist	1.00	1.00	-
Nurse	3.28 (2.25; 4.79)	2.80 (1.80; 4.35)	<0.001
Outras	0.37 (0.15; 0.88)	0.44 (0.17; 1.11)	0.083
**Level of higher education**			
Master’s degree/doctorate	1.00	1.00	-
Specialization/residency	2.19 (1.33; 3.61)	1.92 (1.08; 3.40)	0.025
Degree	2.69 (1.57; 4.59)	2.51 (1.34; 4.70)	0.004
Time working in the Brazilian National Health System (years)	1.04 (1.02; 1.06)	1.04 (1.01; 1.06)	0.004
**Main form of access to the course**			
Computer	1.00	1.00	-
Cell phone or tablet	2.85 (1.79; 4.54)	1.91 (1.09; 3.34)	0.024
**Prior participation in professional qualification on promoting adequate and healthy eating**			
No	1.00	1.00	-
Yes	1.35 (0.99; 1.83)	1.68 (1.15; 2.46)	0.007
**Prior use of the materials presented during the course**			
Yes	1.00	1.00	-
No	1.43 (0.96; 2.14)	1.81 (1.09; 2.98)	0.021

## Discussion

One third of students dropped out of professional qualification activities aimed at promoting adequate and healthy eating. The odds of dropout were greater among nurses, health professionals from the North and Northeast macroregions, those with a lower level of higher education, longer experience in the SUS, those who accessed the course mainly via cell phone or tablet, those who had previously participated in professional qualification on the theme and those had not previously used the materials presented during the course. 

Distance education is a powerful and viable strategy for SUS personnel learning and qualification, as it reaches a large number of students, does not require traveling and reduces costs. It allows courses to fit in with working hours, with good acceptance by health professionals (6), and contributes to the democratization of access to continuing education actions (9). However, it has challenges, mainly related to the high prevalence of dropout. 

Studies have analyzed distance learning courses offered by the SUS Open University and identified an average dropout rate of 30.9%, with similarities in relation to associated factors, such as difficulties in reconciling study and work demands (7,15,16). 

Other possible determinants of dropout include aspects related to:

- students (gender, age, marital status, family income, lack of time, limited time and technology management skills, level of training, prior knowledge on the subject, mental health conditions, among others) (10,12);
- access to course platforms and to the courses themselves (design, layout, space for interaction, support, suitability for various types of electronic devices, internet access instability, relevance of topics for professional practice, format of assessment activities, quality of teaching resources and not including videos as learning strategies) (10,11,17); and
- working conditions (lack of institutional support for doing courses and lack of infrastructure for access at work) (7,10), reiterating the complexity involved in determining dropout from distance learning courses.

This study found greater odds of not completing the course in the North and Northeast macroregions, which are considered to be the most vulnerable and to have the greatest health system overload (18), including shortage of health professionals (19). These results suggest that the Ministry of Health strategy of prioritizing enrollment in these macroregions, together with the Midwest macroregion, was not sufficient to promote equity regarding course retention and completion, thus demanding structural and training actions (4,20).

Greater likelihood of dropout was also found among nurses, who played a crucial role in facing the COVID-19 pandemic, this being a situation experienced by Classes 2 and 3, which may have made it difficult to undertake continuing education activities (21). During the pandemic, collective actions to promote adequate and healthy eating were suspended. This may have led these professionals to direct their efforts towards continuing health education actions aimed at the COVID-19 health emergency. 

Many health professionals have the perception that actions to promote adequate and healthy eating are the responsibility of nutritionists, this being the main professional category enrolled in and completing the course. Nevertheless, doctors, nurses, as well as other health professionals, are also essential for promoting adequate and healthy eating via the SUS, especially in Primary Health Care, as they are the professionals who have initial contact with SUS service users (4). 

Dropout from the course was also higher among those with a lower level of higher professional education and longer experience in the SUS. Professionals with these characteristics may have been trained for longer and in another context of health education, in addition to possibly having greater responsibilities at work and greater difficulties in starting studying again, especially online (22). Professionals with a higher level of higher education and less time working in the SUS tend to look for ways to boost their curriculum vitae and career, in addition to being more familiar with online learning platforms, which can facilitate completion of the course (4). 

Using cell phones or tablets to access the course was also more prevalent among those who dropped out. Health professionals pointed to technological difficulties among the main reasons for dropout. Distance learning courses may not be intuitive on mobile devices, as many of these courses are developed for computers, thus causing greater difficulties (17). This result points to an important issue: the possible unavailability of computers and internet connections in the workplace or at home. Although 93.3% of primary health care centers in Brazil have internet access, almost a quarter of them have inadequate internet connection (23). It is crucial to ensure adequate infrastructure and a work schedule with time allocated to encourage the completion of professional qualification activities and avoid the reproduction of social and cultural inequalities in the work context (20). 

Students who had previously completed professional qualification courses on promoting adequate and healthy eating had a higher rate of dropout. This may have occurred due to less motivation as they may have considered that they had already acquired this knowledge, or due to less motivation to specifically search for information, materials or modules (20,24). In the case of Classes 2 and 3, the COVID-19 pandemic may have led professionals to prioritize continuing education actions on the pandemic. 

Not having previously used the materials for promoting adequate and healthy eating presented during the course was also a negative factor. This showed that, even with prior knowledge on the topic, it is important that instructional materials reach health professionals so that continuing education activities have greater practical significance (4). Previous contact with the materials used in the courses can generate greater interest and promote familiarity with the topics discussed. 

In the perception of those who did not complete the course, lack of time was the main reason for dropping out, with the COVID-19 pandemic also being important for Classes 2 and 3. Lack of time has been highlighted as a significant challenge among SUS professionals (25,26) due to high work demands and physical and mental overload (27). The COVID-19 pandemic generated more exhausting and stressful work activities, often resulting in overload and lack of time for continuing education (28,29). This was identified more among females, who made up the majority of health professionals working on the front line (30), in addition to household chores (9,25) and childcare due to school closures during the pandemic (30).

Addressing distance learning courses dropout rates is a challenge that requires integrated actions among the three federative levels of government. Since the publication of the second edition of the Food Guide (2), the Ministry of Health has intensified production of instructional materials and provision of online professional qualification activities (3,4,13,14). There is an urgent need to define effective strategies that increase access to and use of these materials in daily work, including those aimed at completing professional qualification activities.

It is crucial to ensure health professionals have time available to undertake continuing health education activities at work, as recommended by the National Policy for Continuing Health Education (5), in addition to adequate infrastructure, such as computers and internet access. Due recognition of continuing health education activities in career progression in the SUS is necessary. It is also important to promote prior access to materials presented during courses, preferably printed, given potential difficulties for professionals in using digital materials due to the scarcity of computers and digital literacy. Additional strategies, such as greater frequency of contact with tutors, flexible deadlines and assessments, and use of dynamic audiovisual resources, may also be useful (7,10,28)

Although this study has moved forward with the investigation of factors associated with dropout from online courses intended to promote adequate and healthy eating, we did not assess other possible factors, such as marital status, number of children, head of family and the presence of incentives for professional qualification in their employment career plan, as well as contextual factors. The need for new studies that investigate the topic in depth stands out in order to expand the body of evidence. Another limitation concerns possible information bias due to the temporality of the questions answered three months after completing the course. This interval was necessary in order to identify possible practical applications of the course in everyday work. 

Despite its limitations, this study is important for informing the actions of municipal, state and federal health service managers as to the design of more successful continuing education activities with higher prevalence of completion. This will enable adequate qualification of health professionals for preventing diseases, promoting health and providing health care for the population. 

The prevalence of dropout from professional qualification activities intended to promote adequate and healthy eating was similar to that found in the literature, with multiple associated factors. The odds of dropout were higher among nurses, those who worked in the North and Northeast macroregions, those with a lower level of professional training and longer experience in the SUS, those who accessed the course mainly via cell phone or tablet, those who had previously undertaken professional qualification activities on the topic and those who had not previously used the materials presented during the course. 

These results suggest that regional inequalities in professional training and infrastructure are challenges for professionals to become more qualified. Qualification on the subject of adequate and healthy eating in the SUS is urgent, given the need to stop the growth of obesity and other chronic diseases. This requires health service managers, at different federative levels of management, to implement strategies that favor health professional access, retention and completion of continuing education actions, such as dedicated time on their agenda for doing professional qualification activities and these actions being aligned with their career plans. 

## Data Availability

The databases, codes, methods and other materials used and resulting from this research can be requested (together with a justification) from the corresponding author.
