# Supplementary material for: Distance professional qualification to promote adequate and healthy eating: factors associated with dropout
Source: Epidemiol Serv Saude. 2025 Aug 8;34:e20240338. doi: 10.1590/S2237-96222025v34e20240338.en (PMC12342728; doi:10.1590/S2237-96222025v34e20240338.en)
Supplement: Translation

# Translation: Qualificação profissional à distância para promoção da alimentação adequada e saudável: fatores associados à evasão

**DOI:** 10.1590/S2237-96222025v34e20240338.pt

**Authors:** Mariana Souza Lopes, Izabela Santana Sathler, Cheila de Sousa Jardim Soares, Nathália Luíza Ferreira, Aline Cristine Souza Lopes

**Affiliations:** 1 Universidade Federal da Paraíba, Centro de Ciências da Saúde, Departamento de Nutrição, PB, Brasil; 2 Universidade Federal de Minas Gerais, Escola de Enfermagem, Departamento de Nutrição, MG, Brasil; 3 Universidade Federal de Lavras, Faculdade de Ciências da Saúde, MG, Brasil

**Keywords:** Sistema Único de Saúde, Alimentação Saudável, Educação à Distância, Educação Continuada, Estudos Transversais

## Abstract

**Objetivo:**

Analisar os fatores associados à evasão de qualificação profissional à distância para promoção da alimentação adequada e saudável no Brasil.

**Métodos:**

Trata-se de estudo transversal com profissionais do Sistema Único de Saúde (SUS) matriculados em curso à distância, ofertado entre 2019 e 2020, pautado em materiais do Ministério da Saúde. Avaliaram-se dados sociodemográficos e profissionais associados à evasão, utilizando regressão logística múltipla (p-value<0,05).

**Resultados:**

A prevalência de evasão foi 30,6%. A evasão se associou a residir nas macrorregiões Norte (p-valor 0,009) e Nordeste (p-value<0,001), ser enfermeiro (p-value<0,001), possuir menores níveis de formação (graduação: p-valor 0,004; especialização/residência: p-valor 0,025), ter maior tempo de atuação no SUS (p-valor 0,004), ter realizado o curso por meio de celular ou tablet (p-valor 0,024), ter qualificação prévia sobre alimentação adequada e saudável (p-valor 0,007) e não ter utilizado anteriormente os materiais do curso (p-valor 0,021).

**Conclusão:**

A evasão se associou a residir entre os enfermeiros, profissionais das macroregiões Norte e Nordeste, com menor nível de formação, maior tempo de atuação no SUS, e entre aqueles que acessaram o curso principalmente por celular ou tablet, que haviam participado previamente de qualificação profissional sobre a temática e que não haviam utilizado os materiais empregados no curso. A modificação desses fatores requer apoio institucional, com integração dos diferentes entes federados, visando superar os desafios locais enfrentados pelos profissionais para a conclusão de atividades de educação permanente.

**Aspectos éticos**

A presente pesquisa respeitou os princípios éticos, obtendo os seguintes dados de aprovação: :

Comitê de ética em pesquisa: Universidade Federal de Minas Gerais

Número do parecer: 4294291

Data de aprovação: 23/9/2020

Certificado de apresentação de apreciação ética: 93992418.0.0000.5149

Registro do consentimento livre e esclarecido: Assinado por todos os participantes.

## Introdução

A promoção da alimentação adequada e saudável está presente em diferentes marcos normativos brasileiros, como a Política Nacional de Alimentação e Nutrição e a Política Nacional de Promoção da Saúde. Consiste em estratégias de incentivo a práticas alimentares biológica e socioculturalmente apropriadas, que contribuam para a melhoria da qualidade de vida, da alimentação e da saúde da população (1). Para basear as ações de promoção da alimentação adequada e saudável no Sistema Único de Saúde (SUS), utiliza-se o *Guia Alimentar para a População Brasileira*, instrumento inovador que considera as diferentes dimensões da alimentação (2). 

Entre os diferentes desdobramentos do guia alimentar que visam à sua implementação no SUS, destacam-se os materiais produzidos pelo Ministério da Saúde (MS): “Instrutivo: metodologia de trabalho em grupos para ações de alimentação e nutrição na Atenção Básica” (3) e seus materiais de apoio. Além de disponibilizar materiais instrucionais, é necessário qualificar os profissionais para sua utilização no trabalho (4). Assim, o MS tem investido em ações de educação permanente em saúde alinhadas às diretrizes do SUS (5).

Realizar ações de educação permanente em saúde em um país continental como o Brasil e no contexto de diferentes realidades do SUS exige estratégias abrangentes e flexíveis, como a educação à distância (6). Entretanto, essa modalidade apresenta limitações, com destaque para os elevados percentuais de evasão, que variaram de 30,0% a 75,0% (7,8). Essas elevadas prevalências de evasão podem acarretar subutilização de recursos materiais e humanos, além de comprometer a qualificação dos profissionais (9), o que destaca a relevância de melhor investigá-las. 

Fatores como suporte técnico e didático insuficiente, dificuldades tecnológicas, qualidade do curso, ausência de interação social, alta carga de atividades no trabalho ou no estudo e satisfação e motivação do aluno estão entre os principais aspectos relacionados à não conclusão de cursos à distância (10,11,12). Os dados desta pesquisa poderão apoiar os gestores e contribuir para o delineamento de estratégias mais robustas e efetivas de permanência e certificação dos alunos. Este estudo objetivou analisar os fatores associados à evasão em atividade de qualificação profissional à distância voltada para a promoção da alimentação adequada e saudável entre profissionais do SUS de todo o país. 

## Métodos

### Delineamento do estudo 

Trata-se de estudo transversal que analisou dados de profissionais de saúde concluintes e não concluintes de atividade de qualificação profissional voltada para o desenvolvimento de atividades coletivas no SUS, denominada “Curso de qualificação profissional para promoção da alimentação adequada e saudável na Atenção Básica”. 

### Contexto

A atividade foi realizada na modalidade à distância entre maio de 2019 e novembro de 2020, totalizando três turmas ofertadas. O curso foi delineado por grupo de pesquisa em parceria com a Coordenação-Geral de Alimentação e Nutrição do Departamento de Prevenção e Promoção da Saúde da Secretaria de Atenção Primária à Saúde do MS, e ofertado via Telessaúde, a partir do ambiente virtual de aprendizagem Moodle. 

O MS solicitou e apoiou a realização da qualificação, sobretudo em razão da necessidade de aperfeiçoar a atuação dos profissionais de saúde do SUS, em especial, na Atenção Primária à Saúde, quanto às ações de promoção da alimentação adequada e saudável.

A atividade foi baseada no *Guia Alimentar para a População Brasileira* (2), no “Instrutivo: metodologia de trabalho em grupos para ações de alimentação e nutrição na Atenção Básica” (3) e em seus materiais de apoio: “Desmistificando dúvidas sobre alimentação e nutrição: material de apoio para profissionais de saúde” (13) e “Na cozinha com as frutas, legumes e verduras” (14).

O projeto político pedagógico do curso foi discutido com pedagogos da equipe do Telessaúde e aprovado pelo MS, constando de seis módulos, sendo 11 videoaulas, cinco vídeos de curta duração, cinco fóruns de discussão e sete atividades práticas. No módulo I, foi apresentado o cenário brasileiro de alimentação e nutrição. No módulo II, discutiu-se o conceito de alimentação adequada e saudável com base no guia alimentar (2). No módulo III, foram abordados os conceitos de educação permanente em saúde e de educação alimentar e nutricional. Os módulos IV, V e VI contemplaram os conteúdos e as aplicações da metodologia de grupos para a promoção da alimentação adequada e saudável (3).

O curso teve carga horária de 30 horas, com duração de, no mínimo, dois meses e meio. Para as turmas 2 e 3, o curso foi estendido para 11 meses devido à pandemia de coronavírus (covid-19). A divulgação ocorreu por vídeo de curta duração veiculado por e-mail, aplicativos de mensagens e redes sociais do grupo de pesquisa e de seus colaboradores, do Telessaúde e do MS. Este também enviou cartas-convite aos gestores estaduais e municipais.

### Participantes

Profissionais de ensino superior da área da saúde, de todas as macrorregiões do país, e que atuassem ou desenvolvessem ações no SUS estavam aptos para se inscrever na atividade de qualificação profissional. Isso incluía nutricionistas, enfermeiros, médicos, psicólogos, assistentes sociais e fonoaudiólogos. 

As inscrições nas turmas 1 e 2 foram primeiramente realizadas nas macrorregiões Norte, Nordeste e Centro-Oeste visando promover a equidade de acesso. As inscrições permaneceram abertas por, aproximadamente, 20 dias e foram validadas segundo o Cadastro Nacional de Estabelecimentos de Saúde. A turma 3 incluiu alunos indicados pelos estados participantes da Chamada para Enfrentamento e Controle da Obesidade no SUS, visando potencializar as ações de promoção da alimentação adequada e saudável no país. 

Os critérios de inclusão para participação neste estudo foram: ser aluno das turmas 1, 2 ou 3, que respondeu ao instrumento de avaliação enviado três meses após o término do curso. Foram excluídos os participantes que não responderam às questões de identificação, como nome completo e e-mail.

### Tamanho do estudo 

Foram analisados todos os alunos inscritos na atividade de qualificação profissional, independentemente da conclusão do curso, e que atenderam aos critérios de inclusão e de exclusão.

### Fontes de dados/medidas

Os dados relativos aos participantes da atividade de qualificação profissional foram obtidos em dois momentos: no início do curso e três meses após o término do curso. No início, aplicaram-se formulário de inscrição e questionário contendo dados sociodemográficos, sobre a formação e atuação profissional, participação prévia em atividades de qualificação profissional sobre a temática e o uso dos materiais empregados no curso (2,3,13,14). 

Após três meses, foi enviado, por e-mail, questionário no formato Google Forms. Enviaram-se até três notificações durante três semanas consecutivas visando obter a resposta. Esse questionário incluiu perguntas relativas à aplicabilidade e à pertinência do conteúdo e dos materiais do curso na prática profissional, à realização de atividades educativas propostas, à disseminação dos materiais e às possíveis barreiras para a conclusão do curso.

### Variáveis 

A variável desfecho deste estudo foi a “evasão da atividade de qualificação profissional”, identificada a partir da comparação entre as listas de emissão de certificados com as listas de alunos inscritos.

As covariáveis analisadas foram as elencadas a seguir.

- Sociodemográficas: sexo (feminino, masculino), idade (anos), faixa etária (anos: 19-30, 31-40, 41-70) e macrorregião (Sudeste, Sul, Centro-Oeste e Distrito Federal, Nordeste, Norte).
- Formação e atuação profissional: profissão (nutricionista, enfermeiro, outra – agente comunitário de saúde, assistente social, graduando, biólogo, cirurgião dentista, profissional de educação física, fisioterapeuta, jornalista, psicólogo, técnico de enfermagem), tempo de conclusão da graduação (anos), nível de formação (graduação, especialização, residência, mestrado, doutorado), tempo de atuação no SUS (anos) e jornada de trabalho (horas/semana).
- Qualificação profissional e acesso a materiais de promoção da alimentação adequada e saudável: principal forma de acesso ao curso (computador, celular ou tablet), participação prévia em atividades de qualificação profissional sobre promoção da alimentação adequada e saudável (não, sim), uso prévio dos materiais empregados no curso (não, sim), turma do curso que participou (1, 2, 3), principal motivo da evasão (falta de tempo, dificuldades operacionais na plataforma, infraestrutura inadequada, aumento da carga horária de trabalho, conteúdo da qualificação, outros, sem obstáculos), e, para as turmas 2 e 3, se a pandemia de covid-19 interferiu na realização do curso (não, sim).

A qualidade do curso não foi avaliada entre os alunos não concluintes, uma vez que essa avaliação era uma das atividades do último módulo da qualificação profissional. Caso o aluno tivesse evadido antes de acessar o módulo em questão, não teria acesso à atividade. A avaliação do curso realizada por alunos que não o concluíram poderia ser enviesada e não equiparada, pois cada um pode ter evadido em um módulo distinto.

### Viés

Para garantia do controle e da qualidade dos dados, e adequada validade interna do estudo, todos os instrumentos foram pré-testados e ajustados conforme as necessidades identificadas. Todo o processo de coleta dos dados foi supervisionado por um profissional de saúde, sob a orientação das pesquisadoras.

### Métodos estatísticos 

As análises foram realizadas no Statistical Software for Data Science Stata 15.0, incluindo análises descritivas, com cálculo das distribuições de frequências, medidas de tendência central e de dispersão. As variáveis categóricas foram apresentadas em frequência relativa, e as contínuas, em mediana e intervalo interquartílico (P_25_ – P_75_). Os dados sobre os alunos não concluintes e concluintes foram comparados pelos testes estatísticos de qui-quadrado de Pearson e Mann-Whitney. As variáveis associadas ao desfecho com nível de significância menor que 20% foram incluídas em modelo de regressão logística múltipla (p-valor<0,05) e apresentadas por razão de chances (*odds ratio*, OR) e intervalos de confiança de 95% (IC95%).

## Resultados

Dos alunos respondentes (n=818), 69,4% (n=568) concluíram o curso, com prevalência de evasão de 30,6% (n=250). Na percepção dos profissionais, a falta de tempo foi o principal obstáculo para a conclusão (78,3%), seguida de dificuldades operacionais com a plataforma virtual (9,7%) e infraestrutura inadequada do local de trabalho (5,5%) (Figura 1). Nas turmas 2 e 3, 38,3% dos alunos apontaram a pandemia de covid-19 como obstáculo à conclusão do curso (Figura 2A), com maior prevalência de mulheres (96,2%), em comparação àqueles que não sofreram interferência da pandemia (91,0%; p-valor 0,028). Não houve diferenças quanto à macrorregião de atuação, à faixa etária, à profissão e ao grau de formação acadêmica dos participantes (p-valor>0,05) (Figuras 2B e 2C).

**Figura 1:** Principais fatores que contribuíram para a evasão do curso de qualificação profissional para promoção da alimentação adequada e saudável na atenção básica. Brasil, 2019-2020 (n=486)

**Figura 2:** Influência da pandemia de covid-19 na evasão do curso de qualificação profissional para promoção da alimentação adequada e saudável na atenção básica, e características sociodemográficas e de formação profissional conforme relato de impacto da pandemia na conclusão do curso. Brasil, 2020 (n=486)

Alunos que evadiram a atividade de qualificação profissional, quando comparados aos concluintes, apresentaram maior mediana de idade (em anos: 36; 31-43 versus 35; 30-40; p-valor 0,009] e maior proporção de profissionais que atuavam nas macrorregiões Nordeste (38,8% vs. 24,1%; p-valor<0,001) e Norte (10,8% vs. 6,0%; p-valor<0,001) (Tabela 1).

**Tabela 1:** Prevalências (%) das características sociodemográficas e profissionais dos alunos do curso de qualificação profissional para promoção da alimentação adequada e saudável na atenção básica. Brasil, 2019-2020 (n=818)

Variável	Total (n=818) (%)	Concluintes (n=568) (%)	Não concluintes (n=250) (%)	p-valor
Sexo				0,248
Feminino	93,8	93,5	94,4	
Masculino	6,2	6,5	5,6	
**Macrorregião do país**				<0,001
Sudeste	42,8	46,7	34,0	
Sul	10,4	10,9	9,2	
Centro-Oeste/Distrito Federal	10,8	12,3	7,2	
Nordeste	28,6	24,1	38,8	
Norte	7,5	6,0	10,8	
Profissão				<0,001
Nutricionista	75,1	79,5	65,0	
Enfermeiro	18,3	12,1	32,5	
Outra categoria profissional	6,6	8,4	2,5	
**Nível de formação**				0,001
Graduação	27,4	25,2	32,4	
Especialização/residência	56,9	56,1	58,7	
Mestrado	14,0	16,2	8,9	
Doutorado	1,7	2,5	0,0	
**Mediana** (**percentis 25**-75)				
Idade	35 (30-41)	35 (30-40)	36 (31-43)	0,009
Formação (anos)	12 (8-17)	12 (7-17)	13 (8-17)	0,083
Atuação no Sistema Único de Saúde (anos)	6 (3-12)	5 (2-11)	8 (3,5-14)	<0,001
Jornada de trabalho (horas/semana)	40 (30-40)	40 (30-40)	40 (30-40)	0,176

As maiores prevalências identificadas entre os alunos que evadiram foram de enfermeiros (32,5% vs. 12,1%; p-valor<0,001), profissionais com graduação (32,4% vs. 25,2%; p-valor 0,001) e especialização/residência (58,7% vs. 56,1%; p-valor 0,001) como grau máximo de escolaridade, e maior mediana de tempo de atuação no SUS (em anos: 8; 3,5-14 vs. 5,0; 2-11; p-valor<0,001), quando comparados aos concluintes (Tabela 1).

A forma preferencial de acesso ao curso foi o computador (90,0%). Houve maior prevalência de uso de celular ou tablet (17,4% vs. 6,9%; p-valor<0,001) entre os alunos que evadiram. A maioria dos participantes já havia realizado qualificações sobre o tema (58,3%) e utilizado os materiais de promoção da alimentação adequada e saudável empregados no curso (85,1%) (Figura 3).

**Figura 3:** Acesso ao curso de qualificação profissional para promoção da alimentação adequada e saudável na atenção básica e formação para promoção da alimentação adequada e saudável. Brasil, 2019-2020 (n=818)

Na análise multivariada, verificaram-se maiores chances de evasão da atividade de qualificação profissional entre os profissionais que atuavam nas macrorregiões Norte (OR 2,40; IC95% 1,25; 4,63; p-valor 0,009) e Nordeste (OR 2,16; IC95% 1,44; 3,24; p-valor<0,001), enfermeiros (OR 2,80; IC95% 1,80; 4,35; p-valor<0,001), com menores níveis de formação (graduação: OR 2,51; IC95% 1,34; 4,70; p-valor= ,004 e especialização/residência: OR 1,92; IC95% 1,08; 3,40; p-valor 0,025), que atuavam há mais tempo no SUS (OR 1,04; IC95% 1,01; 1,06; p-valor 0,004), que acessaram o curso preferencialmente pelo celular ou tablet (OR 1,91; IC95% 1,09; 3,34; p-valor 0,024), que participaram previamente de qualificação para promoção da alimentação adequada e saudável (OR 1,68; IC95% 1,15; 2,46; p-valor 0,007) e que não haviam utilizado anteriormente os materiais empregados no curso (OR 1,81; IC95% 1,09; 2,98; p-valor 0,021) (Tabela 2). 

**Tabela 2:** Razão de chances (*odds ratio*, OR) e intervalo de confiança de 95% (IC95%) brutas e ajustadas gerados em análise multivariada dos fatores associados à evasão ao curso de qualificação profissional para promoção da alimentação adequada e saudável na atenção básica. Brasil, 2019-2020 (n=818)

Variáveis	OR bruta (IC95%)	OR ajustada (IC95%)	p-valor
**Macrorregião do país**			
Sudeste	1,00	1,00	-
Sul	1,16 (0,68; 1,98)	1,30 (0,72; 2,35)	0,381
Centro-Oeste/Distrito Federal	0,80 (0,45; 1,42)	0,78 (0,40; 1,51)	0,465
Norte	2,46 (1,41; 4,34)	2,40 (1,25; 4,63)	0,009
Nordeste	2,21 (1,54; 3,15)	2,16 (1,44; 3,24)	<0,001
Profissão			
Nutricionista	1,00	1,00	-
Enfermeiro	3,28 (2,25; 4,79)	2,80 (1,80; 4,35)	<0,001
Outras	0,37 (0,15; 0,88)	0,44 (0,17; 1,11)	0,083
**Nível de formação**			
Mestrado/doutorado	1,00	1,00	-
Especialização/residência	2,19 (1,33; 3,61)	1,92 (1,08; 3,40)	0,025
Graduação	2,69 (1,57; 4,59)	2,51 (1,34; 4,70)	0,004
Atuação no Sistema Único de Saúde (anos)	1,04 (1,02; 1,06)	1,04 (1,01; 1,06)	0,004
**Principal forma de acesso ao curso**			
Computador	1,00	1,00	-
Celular ou tablet	2,85 (1,79; 4,54)	1,91 (1,09; 3,34)	0,024
**Participação anterior em qualificação sobre promoção da alimentação adequada e saudável**			
Não	1,00	1,00	-
Sim	1,35 (0,99; 1,83)	1,68 (1,15; 2,46)	0,007
**Uso anterior dos materiais utilizados no curso**			
Sim	1,00	1,00	-
Não	1,43 (0,96; 2,14)	1,81 (1,09; 2,98)	0,021

## Discussão

Um terço dos alunos evadiu da atividade de qualificação profissional para promoção da alimentação adequada e saudável. As chances de evasão foram maiores entre enfermeiros, profissionais das macrorregiões Norte e Nordeste, com menor nível de formação, maior tempo de atuação no SUS, entre aqueles que acessaram o curso principalmente por celular ou tablet, que haviam participado previamente de qualificação profissional sobre a temática e que não haviam utilizado os materiais empregados no curso. 

A educação à distância é estratégia potente e viável para aprendizagem e qualificação de profissionais do SUS por atingir grande contingente de alunos, não demandar deslocamentos e reduzir os custos. Permite conciliar os cursos com a jornada de trabalho, com boa aceitação dos profissionais de saúde (6), e contribui para a democratização do acesso às ações de educação permanente (9). No entanto, possui desafios, principalmente relacionados às elevadas prevalências de evasão. 

Foram analisados cursos à distância ofertados pela Universidade Aberta do SUS e identificou-se a prevalência média de evasão de 30,9%, com semelhanças em relação aos fatores associados, como as dificuldades em conciliar demandas de estudo e de trabalho (7,15,16). 

Outros possíveis determinantes da evasão incluem aspectos relacionados:

- aos alunos (sexo, idade, estado civil, renda familiar, indisponibilidade de tempo, habilidades de gerenciamento do tempo e das tecnologias limitadas, grau de formação, conhecimentos prévios sobre a temática, condições de saúde mental, entre outros) (10,12);
- às plataformas de acesso e aos cursos em si (design, layout, espaço para interação, apoio e suporte, adequação a distintos equipamentos eletrônicos, instabilidade de acesso à internet, relevância dos temas para a prática, formato das atividades avaliativas, qualidade dos recursos didáticos e não inclusão de vídeos como estratégias de aprendizagem) (10,11,17); e
- às condições de trabalho (falta de apoio institucional para realizar os cursos e ausência de infraestrutura para acesso no trabalho) (7,10), reiterando a complexidade envolvida na determinação da evasão a cursos à distância.

Neste estudo, foram observadas maiores chances de não conclusão nas macrorregiões Norte e Nordeste, consideradas as mais vulneráveis e com maior sobrecarga do sistema de saúde (18), incluindo a escassez de profissionais (19). Esses resultados sugerem que a estratégia do MS de priorizar as inscrições nessas macrorregiões, junto com o Centro-Oeste, não foi suficiente para promover a equidade na permanência e conclusão no curso, demandando ações estruturais e de formação (4,20).

Também foram constatadas maiores chances de evasão entre os enfermeiros, que apresentaram papel crucial no enfrentamento da pandemia de covid-19, situação vivenciada pelas turmas 2 e 3, o que pode ter dificultado a realização de atividades de educação permanente (21). As ações coletivas de promoção da alimentação adequada e saudável foram suspensas. Isso pode ter levado esses profissionais a direcionarem seus esforços para ações de educação permanente em saúde voltadas para a emergência sanitária da covid-19. 

Muitos profissionais possuem a percepção de que as ações de promoção da alimentação adequada e saudável são de responsabilidade do nutricionista, principal categoria profissional inscrita e concluinte do curso. Contudo, médicos, enfermeiros, assim como demais profissionais, são fundamentais para a promoção da alimentação adequada e saudável no SUS, especialmente na Atenção Primária à Saúde, por serem os profissionais de primeiro contato com os usuários (4). 

A evasão do curso também foi maior entre aqueles com menor nível de formação profissional e ao maior tempo de atuação no SUS. Profissionais com essas características, possivelmente, são formados há mais tempo e em outro contexto da educação em saúde, além de possivelmente possuírem maiores responsabilidades no trabalho e maiores dificuldades para a retomada dos estudos, sobretudo à distância (22). Profissionais com maior nível de formação e menor tempo de atuação no SUS tendem a buscar meios de impulsionar o currículo e a carreira, além de estarem mais familiarizados com as plataformas virtuais de aprendizagem, o que pode facilitar a conclusão no curso (4). 

O uso do celular ou de tablet para acessar o curso também foi mais prevalente entre aqueles que evadiram. Profissionais de saúde apontaram as dificuldades tecnológicas entre os principais motivos para evasão. Os cursos à distância podem não ser intuitivos em aparelhos móveis, visto que boa parte é desenvolvida para computadores, acarretando maiores dificuldades (17). Esse resultado aponta para uma questão importante: a possível indisponibilidade de computadores e de conexão à internet no ambiente de trabalho ou no domicílio. Embora 93,3% das unidades básicas de saúde no Brasil possuam acesso à internet, em quase um quarto a conexão é inadequada (23). É crucial garantir infraestrutura adequada e agenda de trabalho protegida visando favorecer a conclusão de atividades de qualificação profissional e evitar a reprodução das desigualdades sociais e culturais no contexto de trabalho (20). 

Alunos que haviam cursado anteriormente qualificações profissionais sobre promoção da alimentação adequada e saudável apresentaram maiores prevalências de evasão. Isso pode ter ocorrido devido à menor motivação por já considerarem o conhecimento apreendido, ou mesmo devido à busca específica por informações, materiais ou módulos (20,24). Para as turmas 2 e 3, a pandemia de covid-19 pode ter levado os profissionais a priorizarem ações de educação permanente sobre o tema. 

Não ter utilizado antes os materiais de promoção da alimentação adequada e saudável empregados no curso também foi um interferente negativo. Isso evidenciou que, mesmo diante de conhecimento prévio sobre o tema, é importante que materiais instrucionais alcancem os profissionais de saúde para que as atividades de educação permanente tenham maior significado prático (4). O contato prévio com os materiais utilizados nos cursos pode gerar maior interesse e favorecer a familiaridade com os temas discutidos. 

Na percepção dos alunos não concluintes, a falta de tempo foi o principal motivo para a evasão, sendo a pandemia de covid-19 também importante para as turmas 2 e 3. A falta de tempo foi apontada como desafio significativo entre os profissionais do SUS (25,26) devido às elevadas demandas de trabalho e à sobrecarga física e mental (27). A pandemia de covid-19 gerou atividades laborais mais exaustivas e estressantes, resultando, muitas vezes, em sobrecarga e falta de tempo para a educação permanente (28,29). Isso foi mais identificado entre as mulheres, que compuseram a maioria dos profissionais de saúde atuantes na linha de frente (30), acumulando o trabalho doméstico (9,25) e o cuidado de crianças com o fechamento das escolas (30).

O enfrentamento da evasão em cursos à distância é um desafio que exige ações integradas entre os três níveis federados. A partir da publicação da segunda edição do guia alimentar (2), o MS intensificou a produção de materiais instrucionais e a oferta de atividades de qualificação profissional à distância (3,4,13,14). É premente o delineamento de estratégias efetivas que ampliem o acesso e a utilização desses materiais no cotidiano de trabalho, incluindo aquelas voltadas para a conclusão das atividades de qualificação profissional.

É crucial assegurar tempo na agenda dos profissionais para a realização de atividades de educação permanente em saúde no trabalho para o trabalho, conforme preconizado pela Política Nacional de Educação Permanente em Saúde (5), além de infraestrutura adequada, como computadores e acesso à internet. Faz-se necessário o devido reconhecimento das atividades de educação permanente em saúde nas progressões de carreira no SUS. Também é importante promover o acesso prévio aos materiais empregados nos cursos, preferencialmente impressos, haja vista potenciais dificuldades dos profissionais para utilizarem materiais digitais devido à escassez de computadores e de letramento digital. Estratégias adicionais, como maior frequência de contato com os tutores, maleabilidade nos prazos e avaliações e uso de recursos audiovisuais dinâmicos, podem ser úteis (7,10,28)

Embora este estudo tenha avançado na investigação dos fatores associados à evasão de cursos de promoção da alimentação adequada e saudável veiculados à distância, outros possíveis interferentes, como estado civil, número de filhos, chefia da família e presença de incentivos para qualificação profissional no plano de carreira, bem como fatores contextuais, não foram avaliados. Salienta-se a necessidade de novos estudos que investiguem em profundidade a temática visando ampliar o corpo de evidências. Outra limitação diz respeito a possível viés de informação devido à temporalidade das questões respondidas três meses após a conclusão do curso. Esse intervalo foi necessário visando identificar possíveis aplicações práticas do curso no cotidiano de trabalho. 

Apesar de suas limitações, este estudo é importante para instrumentalizar a ação de gestores municipais, estaduais e federais para o delineamento de atividades de educação permanente mais exitosas e com maiores prevalências de conclusão. Isso possibilitará a adequada qualificação dos profissionais para a promoção, prevenção e assistência à saúde da população. 

A prevalência de evasão de atividade de qualificação profissional para a promoção da alimentação adequada e saudável foi semelhante à literatura, sendo múltiplos os fatores associados. As chances de evasão foram superiores entre os enfermeiros, que atuavam nas macrorregiões Norte e Nordeste, com menor grau de formação profissional e maior tempo de atuação no SUS, que acessaram o curso principalmente pelo celular ou tablet, que tinham realizado anteriormente atividade de qualificação profissional sobre o tema e que não haviam utilizado antes os materiais empregados no curso. 

Esses resultados sugerem que desigualdades regionais na formação profissional e na infraestrutura são desafios para os profissionais se qualificarem. A qualificação sobre a temática no SUS é urgente, tendo em vista a necessidade de deter o crescimento da obesidade e de demais doenças crônicas. Isso demanda dos gestores, nos diferentes níveis federados, a implementação de estratégias que favoreçam o acesso, a permanência e a conclusão dos profissionais de saúde em ações de educação permanente, como o horário protegido na agenda para realização de atividades de qualificação profissional e o alinhamento aos planos de carreira. 

## Data Availability

Os bancos de dados, códigos, métodos e outros materiais utilizados e resultantes da pesquisa podem ser solicitados à autora correspondente, mediante justificativa.
